# Echocardiography in acute stroke patients: a nationwide analysis in departments with certified stroke units in Germany

**DOI:** 10.1186/s42466-022-00229-1

**Published:** 2023-01-19

**Authors:** Timolaos Rizos, Ekkehart Jenetzky, Darius Günther Nabavi, Karl Georg Haeusler, Rolf Wachter, Martin Ossenbrink, Peter Arthur Ringleb, Otto Busse

**Affiliations:** 1 Department of Neurology, University of Heidelberg, Im Neuenheimer Feld 400, 69120 Heidelberg, Germany; 2 Faculty of Health/School of Medicine, Witten/Herdecke University, Witten, Germany; 3 Department for Child and Adolescent Psychiatry, Johannes Gutenberg-University, Mainz, Germany; 4 Department of Neurology, Vivantes Klinikum Neukölln, Rudower Str 48, 12351 Berlin, Germany; 5 Department of Neurology, University of Würzburg, Würzburg, Germany; 6 Department of Cardiology, University Hospital Leipzig, Leipzig, Germany; 7 LGA InterCert GmbH, Nuremberg, Germany; 8 Deutsche Schlaganfall Gesellschaft, Berlin, Germany

**Keywords:** Ischemic stroke, TIA, Echocardiography, Stroke unit, Guidelines

## Abstract

**Background:**

Echocardiography is highly relevant in patients with ischemic stroke or TIA. Utilization of routine echocardiographic examinations [transthoracic (TTE) or transesophageal (TEE)] on stroke units remains however unknown. To representatively examine echocardiographic rates on stroke units in Germany and to evaluate structural factors that may influence the decision to conduct echocardiography.

**Methods:**

A nationwide analysis was performed by using certification audit data of all primary and comprehensive stroke centers (pSC and cSC) in Germany.

**Results:**

Structural and organizational requirements of 310 departments (cSCs: 42.6%) were extracted. Median TTE rate was 63.3% (IQR 39.3–80.8), median TEE rate 21.3% (IQR 16.4–29.5). A cardiological department on site was present in 74.2%, and they were associated with higher TEE rates. TTE rates decreased with increasing numbers of patients (*p* = 0.026). Likewise, TEE rates decreased with increasing numbers of patients (*p* = 0.006), mediated by departments with cSCs (*p* = 0.008 for cSCs vs *p* = 0.230 for pSCs). TTE rates were far more inhomogeneously distributed than TEE rates and higher in pSCs (*p* = 0.011). Overall, 12.9% of centers did not perform any echocardiographic examination in at least 50% of all stroke patients.

**Conclusion:**

More detailed recommendations regarding echocardiography should be included in future guidelines. Moreover, evaluating the impact of echocardiographic examinations on long-term prognosis in stroke patients should be focus of further evaluations.

**Supplementary Information:**

The online version contains supplementary material available at 10.1186/s42466-022-00229-1.

## Introduction

Echocardiography is essential to evaluate cardiac function, to diagnose heart disease and to detect embolic sources of stroke in patients with acute ischemic strokes or transient ischemic attack (TIA). By using different modalities, specifically transthoracic (TTE) or transesophageal (TEE) echocardiographic examinations, it can detect possible embolic sources of stroke and additionally provides crucial information on structural and functional heart disorders, which e.g. includes left ventricular dysfunction in patients with covered arterial hypertension [1, 2]. Moreover, diseases of the ascending aorta, including major aortic plaques or dissections, can be detected by this method, which is considered to be cost-effective [3, 4]. Echocardiographic results can help to identify probable causes of ischemic stroke or TIA [3]. They may have instant therapeutic implications (e.g. in case of endocarditis) in stroke patients, or can result in changes of secondary preventive measures [3] but rather high numbers of examinations are needed to detect one treatment relevant findings in observational studies [5]. Of note, echocardiography is also able to identify ischemic stroke or TIA patients prone to future major cardiovascular diseases, including heart failure, myocardial infarction, and vascular death [6]. Echocardiography may moreover guide important further diagnostic procedures, including long-term ECG monitoring to detect paroxysmal atrial fibrillation in patients with left atrial pathology [3, 7–10].

Taken together, echocardiography is highly relevant in patients with ischemic stroke or TIA. However, there is no randomized controlled trial demonstrating a reduction of clinically relevant endpoints in stroke patients undergoing echocardiography [3] and as a result, recommendations regarding the extend of echocardiographic examinations in stroke patients are based on expert consensus and current guidelines remain vague and inconsistent [2, 3, 11–14]. It is therefore not surprising, that rates and indications for echocardiography on stroke units vary substantially between departments, leading into the impression that it may be performed to some extend arbitrarily. This is supported by findings of a secondary analysis of the Find-AF_RANDOMISED_ trial evaluating cardiac pathologies detected by echocardiography in patients with acute ischemic stroke [15]. Here, rates for the different modalities differed enormously between participating stroke centers (TTE only: 2–23%; TEE only: 1–10%, both modalities: 1–13%) [15]. However, it can be supposed that reported numbers in this study do not reflect frequencies of routine echocardiographic examinations on certified stroke units [15]. To close this relevant knowledge gap, we aimed to representatively examine rates of TTE and/or TEE in departments with certified stroke units in Germany, and to evaluate structural factors that may influence the decision to conduct echocardiography after stroke or TIA.

## Patients and methods

We performed a nationwide cross-sectional analysis including departments with a certified stroke unit in Germany [16]. The stroke unit certification scheme aims to guarantee high quality stroke care throughout Germany. This system includes a multilevel medical care approach, including primary stroke centers (pSC), comprehensive stroke centers (cSC) and tele-stroke units. To obtain certification by the German Stroke Society, among other obligations, a minimum of stroke patient volume, predefined interventions, quantified staff level resources, and specific training obligations for the respective medical team are required [16]. Certification prerequisites also include a minimum TEE rate of 15% in stroke patients whereas no TTE rates are specified up to now [16]. Certificates are granted for 3 years after passing a local audit on site, done by a stroke expert.

For the present study, we performed a secondary analysis of audit data regarding utilization of echocardiography in stroke patients. Due to their limited number, varying structural prerequisites and certification-criteria and overall limited comparability to pSCs and cSCs, tele-stroke-units were not included into the present analysis. To obtain a representative dataset, one “audit cycle” between 1st January, 2018 and 31th December, 2020 was used. By using this approach, the present analysis contains almost all certified stroke units in Germany, covering the vast majority of all hospital admissions due to stroke in Germany.

We recorded basic structural and organizational data, including the respective hospital type (university hospital or non-university hospital), stroke unit type (pSC or cSC), and information on the presence of an independent neurological and/or cardiological department. Moreover, numbers of stroke unit beds per department and, numbers of stroke patients [including ischemic stroke, TIA and primary intracerebral hemorrhage (ICH)] treated per department and year were extracted from audit reports of each center. Furthermore, reported numbers of TTEs and TEEs in ischemic stroke and TIA patients per department were extracted. Using these items, additional variables to describe patterns of echocardiographic examinations and structural factors potentially influencing the respective rates were derived. Departments with incomplete or implausible TTE and/or TEE rates were excluded.

All audits are executed by the LGA InterCert GmbH, Nuremberg, Germany and by stroke experts serving as auditors in a structured and predefined manner on-site. Data are collected, processed and stored by this body according to German and European data protection law. While no individual patient data are recorded, patient consent was not required. Data extracting authors of the present study (OB, TR) signed a secrecy declaration of the LGA InterCert GmbH. All extracted data were anonymized for analysis and subsequent presentation. The manuscript was developed according to GPS guidelines for reporting secondary data [17]. This study was performed in line with the principles of the Declaration of Helsinki. The local ethics committee of the Medical Faculty of Heidelberg University approved the study. Because of the study character, patients’ consent was waived.

### Statistical analysis

Descriptive data are presented in absolute and relative frequencies, ordinal and continuous data as medians and interquartile ranges (IQR). Differences between groups were examined by using univariate nonparametric tests. To evaluate relations between TTE and TEE rates, TTE/TEE rates and type of stroke unit and departmental numbers of stroke patients, linear logistic regression analyses were performed. To assess associations between TTE/TEE rates and the existence/nonexistence of a cardiological department, rates of echocardiographic examinations were additionally partitioned into quartiles. Moreover, for each department we calculated rates of patients in whom no echocardiography or combined methods (TTE and TEE) were performed. We assumed that one TTE and/or one TEE was done per single patient, and applied the following formula: (TTErate + TEErate)-100. A two-sided *p* value of < 0.05 was considered explorative significant. Data were analyzed by using the Statistical Package for the Social Sciences (SPSS 28.0).

## Results

### Data set and basic organizational information

Within the study period of 36 months, a total of 341 departments with a stroke unit underwent a certification audit. As predefined, tele-stroke-units (N = 21) and centers reporting incomplete or implausible TTE or TEE rates were excluded (N = 10). Therefore, the final data set included 310 departments. Of these, 132 departments (42.6%) cSCs and 178 (57.4%) a pSCs. Nearly all stroke units were embedded into a neurological department (97.1%, N = 301). A cardiological department in the respective hospitals was present in 74.2% (N = 230). Reported and calculated variables with regard to the type of the stroke units and respective differences are displayed in Table 1. No significant change of echocardiographic numbers and rates were present between the 3 years of the audit cycle (Additional file 1: Table S1).

**Table 1:** Reported and calculated variables of all included departments, with regard to the type of stroke units and respective differences

Variable	All included stroke units (N = 310)	Comprehensive stroke centers (N = 132; 42.6%)	Primary stroke centers (N = 178; 57.4%)	*p*
*Reported variables (included departments)*				
University hospital: N (%)	31 (10.0)	30 (22.7)	1 (0.6)	**< 0.001**^**a**^
Neurological department present: N (%)	301 (97.1)	132 (100)	169 (94.9)	**0.012**^**a**^
Cardiological department present: N (%)	230 (74.2)	117 (88.6)	113 (63.5)	**< 0.001**^**a**^
Number of stroke unit beds: N (IQR)	8 (4–22) IQR: 6–10	10 (8–12)	6 (5–8)	**< 0.001**^**b**^
Stroke patients per department: median (IQR)^c^	784 (583–1043)	1039 (791–1291)	654 (480–845)	**< 0.001**^**b**^
Number of TTEs per department and year: median (IQR)	455 (269–645)	552 (371–777)	401 (243–586)	**< 0.001**^**b**^
Number of TEEs per department and year: median (IQR)	170 (121–243)	216 (159–303)	140 (102–200)	**< 0.001**^**b**^
*Calculated variable (included departments)*				
TTE rate (%) per stroke patients: median (IQR)	63.3 (39.0–80.8)	57.9 (33.4–76.8)	69.8 (43.3–84.3)	**0.008**^**b**^
TEE rate (%) per stroke patients: median (IQR)	21.7 (16.4–29.5)	21.5 (16.5–29.2)	20.9 (16.4–30.0)	0.733^b^
Rates of patients with no-, or combined examinations (%) [TTE rate + TEE rate) − 100]: median (IQR)	− 13.6 (− 33.5 to 5.1)	− 19.7 (− 40.3 to − 1.9)	− 8.7 (− 27.9 to 8.3)	**0.002**^b^

Bold indicates results were statistically significant

*TTE* transthoracic echocardiography, *TEE* echocardiography, *N* = number

^a^Chi^2^-Test

^b^Kruskal–Wallis Test; *IQR* Interquartile range

^c^including ischemic stroke, TIA and primary intracerebral hemorrhage (ICH) patients

Overall, 260.790 hospitalized stroke patients [median per department: 784 patients (IQR 583–1043; range: 252–2.229)] were treated at 2.604 stroke unit beds [median per stroke unit: 8 beds (IQR 6–10; range: 4–22)].

### TTE rates

**Fig. 1:**
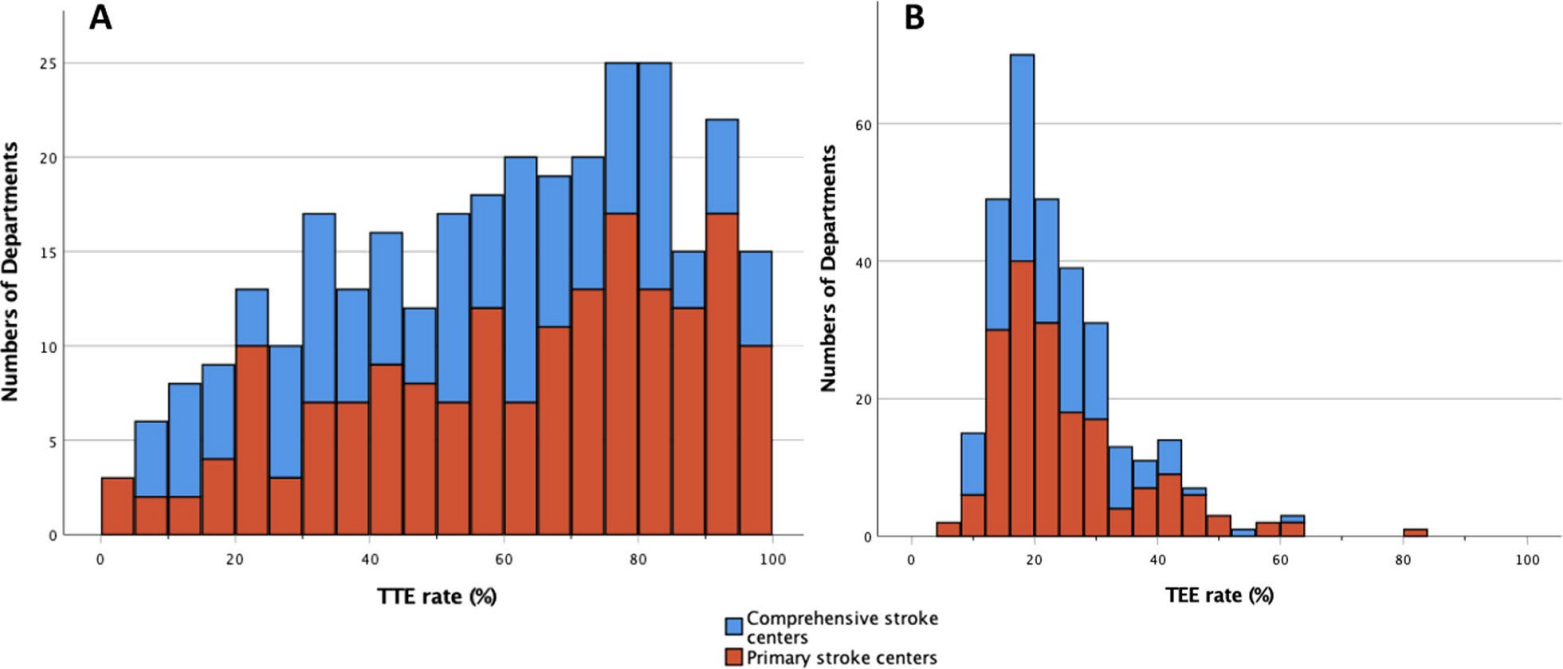
**A** Bar chart for TTE rates per patients for primary and comprehensive stroke centers. **B** Bar chart for TEE rates for primary and comprehensive stroke centers

Audit data revealed a total of 152,802 TTEs during the study period. The median number of TTEs per department and year was 455 (IQR 269–645), resulting into a median TTE rate of 63.3% (IQR 39.3–80.8). As also shown in Fig. 1A, TTE rates per department were inhomogeneously distributed. TTE rates were considerably higher in departments with pSCs than in cSCs (median 69.8% (IQR 43.3–84.3) vs. 57.9% (IQR 33.4–76.8); *p* = 0.011). Overall, no association between TTE rate and the presence of a local cardiological department was found (median 63.1% (IQR 38.3–80.4) with vs. 67.3% (IQR 42.9–82.2) without cardiological department; *p* = 0.373). Partitioning TTE rates into quartiles did not reveal an association between TTE rate and the presence of a cardiological department (Additional file 1: Table S2A).

### TEE rates

Audit records included of a total of 61,233 TEE examinations during the study period. The median number of TEEs per department and year was 170 (IQR 121–243), resulting into a median TEE rate of 21.3% (IQR 16.4–29.5). TEE rates were more homogenously distributed than TTE rates (Fig. 1B). TEE rates were similar between departments with comprehensive and primary stroke centers (median 21.5% (IQR 16.5–29.2) vs. median 20.9% (IQR 16.4–30.0); *p* = 0.306). No difference was obvious with regard to the presence of a cardiological department (median rate: 21.5% (IQR 16.2–29.4) with vs 20.1% (IQR 16.5–30.5) without cardiological department; *p* = 0.685). However, partitioning TEE data into quartiles revealed that departments with cSCs and pSCs with highest TEE rates more often had cardiological departments on site (*p* = 0.026 and *p* = 0.042, respectively; Additional file 1: Table S2B).

### Relations between TEE and TTE

**Fig. 2:**
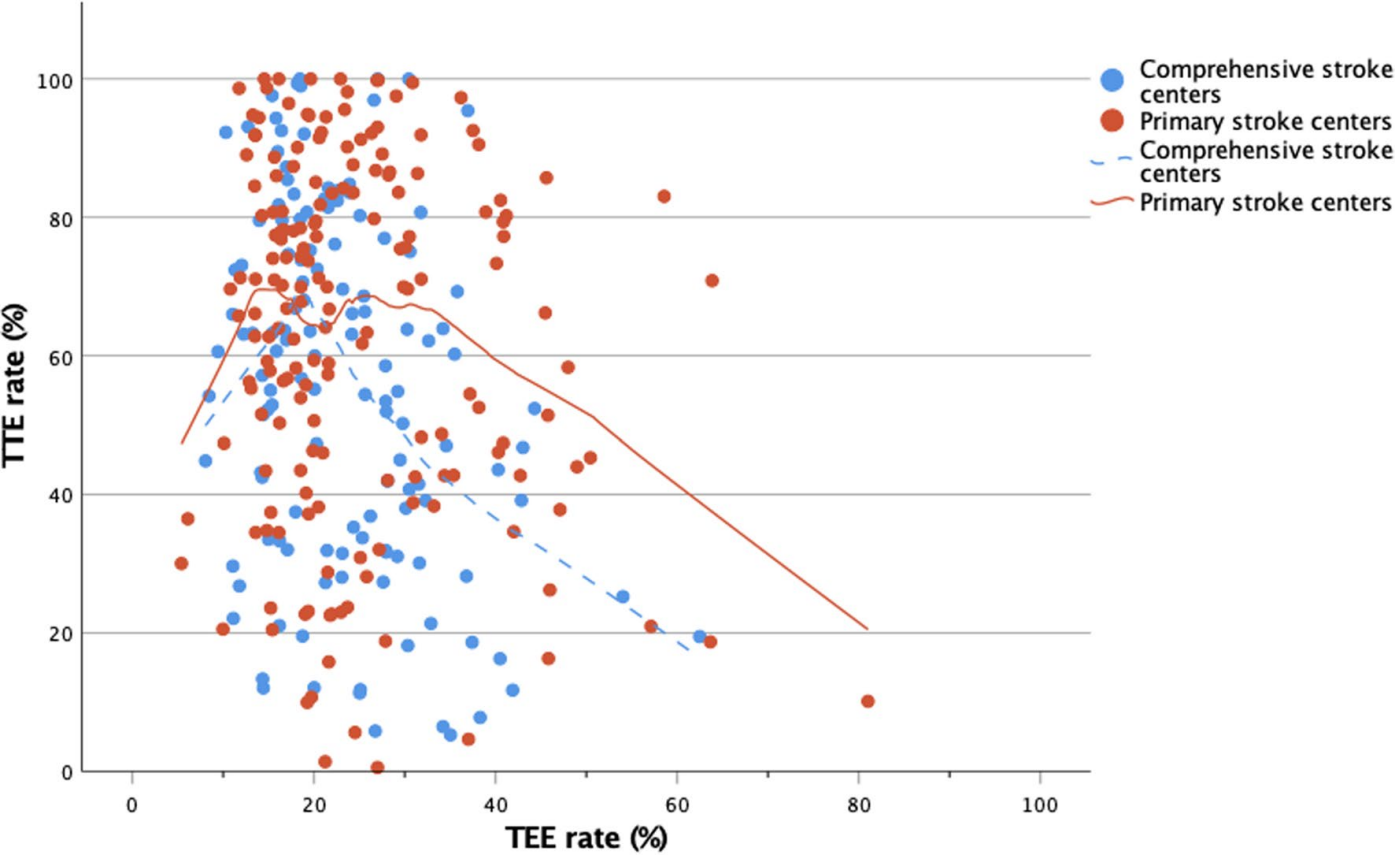
TTE rates in relation to TEE rates per department. Higher TEE rates resulted into lower TTE rates and departments with primary stroke centers had higher TTE rates than departments with comprehensive stroke centers. Both effects can be observed in particular above TEE rates of 20%

Linear regression analysis revealed that higher TEE rates resulted into lower TTE rates (OR − 0.523, 95% CI − 0.785 to − 0.261; *p* < 0.001, Fig. 2). Again, departments with pSCs had higher TTE rates than departments with cSCs (OR 8.061, 95% CI 2.080–14.042; *p* = 0.008). Both effects were observed in particular above TEE rates of 20%. In centers with lower TEE rates, also lower TTE rates were documented (Fig. 2).

No relation between length of hospital stay and TTE rates (OR − 0.273, 95% CI − 1.924 to 1.378; *p* = 0.745) and TEE rates respectively (OR 0.303, 95% CI − 0.328 to 0.988; *p* = 0.385) were observed.

### Echocardiographic examinations and numbers of stroke patients

Linear regression analysis revealed that TTE rates decreased with increasing numbers of stroke patients per department (OR − 0.009; 95% CI − 0.018 to 0.001; *p* = 0.026). This observation was not affected by the type of stroke unit (pSC: OR − 0.003; 95% CI − 0.019 to 0.013; *p* = 0.694 vs. cSC: OR − 0.006; 95% CI − 0.019 to 0.007; *p* = 0.338).

TEE rates decreased with increasing numbers of stroke patients per department (OR − 0.005, 95% CI − 0.008 to − 0.001; *p* = 0.006), mediated by departments with cSCs: (OR − 0.006, 95% CI − 0.011 to − 0.002; *p* = 0.008 vs. departments with pSCs: OR − 0.004, 95% CI − 0.012 to 0.003; *p* = 0.230).

### No-, and combined echocardiographic examinations

**Fig. 3:**
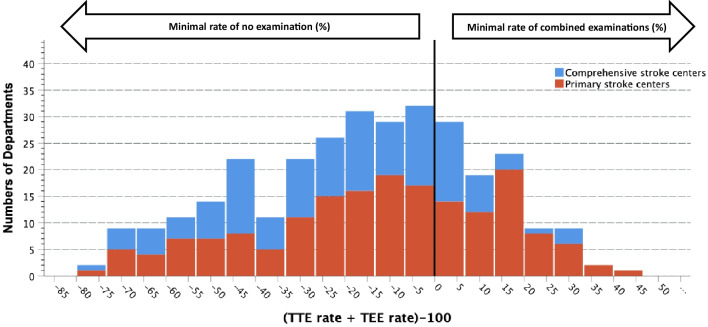
Rates of patients undergoing no-, or combined echocardiographic examinations and numbers of stroke units for which these rates were observed. (For interpretation: − 100: In all patient no examination was performed at all; − 75: in at least 75% of patients no examination was performed; 0: In all patients one examination has been performed; + 10: In at least 10% of patients combined examinations have been done; + 100 = In all patients combined examinations have been done)

As illustrated in Fig. 3, 92 (29.7%) of 310 departments performed combined echocardiographic examinations at all. No significant association between combined examinations and the presence of a local cardiological department was present (data not shown; *p* = 0.777). Combined examinations were more often present in departments with pSCs than in departments with cSCs (68.5% (63/92) vs. 31.5% (29/92); *p* = 0.012; Fig. 3). Overall, 12.9% of centers (40/310) did not perform any echocardiographic examination in at least 50% of all stroke patients and in one single center at least 77.4% of patients had no echocardiographic examination at all.

## Discussion

Against the background of limited evidence regarding echocardiographic imaging in ischemic stroke and TIA patients and the need of optimal allocation of human and financial resources, there is an intense debate to what extend echocardiography should be performed in acute stroke or TIA patients.

To the best of our knowledge, nationwide rates of echocardiographic examinations during routine treatment of acute stroke patients have not been published by now. According to our representative findings, substantial heterogeneity regarding rates and type of echocardiography in stroke patients is present in Germany. Of note, one out of seven stroke units performed no echocardiographic examination in at least 50% of all stroke patients. Not surprisingly, stroke units with highest TEE rates more often had a cardiological department on site. With increasing numbers of stroke-patients treated per year, the proportion of TTE and TEE-examinations declined. Regarding TEE, this finding was driven by cSCs. Finally, TTE rates were higher in pSCs, if compared to cSCs.

Another illustrative finding of the present study is the observation that TTE rates are far more heterogeneously distributed than TEE rates. Certification prerequisites in Germany includes a minimum TEE rate of 15% in ischemic stroke patients [16] and no quorum for TTE. It can be assumed that this demand substantially contributes to our results on TEE use. The highly distributed TTE rates among departments may on the other hand reflect the indefinite statements of current guidelines [2, 3, 11–14]. Notably, ESO guidelines on management of TIA patients and ESO criteria for certification of stroke units do not give any statement with regard to echocardiographic examinations [18, 19].

Inhomogeneously distributed TTE rates may on the other hand also reflect different perceptions of the value of echocardiography in acute stroke patients at the respective centers. Frequently, echocardiography is perceived to detect cardioembolic sources of ischemic stroke or TIA only and most studies focused on direct changes of secondary stroke prevention only [5, 13]. However, echocardiographic findings are also able to identify ischemic stroke and TIA patients prone to future major adverse cardiovascular events, even in case that no explicit embolic source of stroke has been detected [6]. Moreover, echocardiographic findings provide information on structural and functional heart disorders [2, 3] which impact long-term survival and subsequently may lead to reduce the cardiovascular long-term risk by initiating lifestyle changes and pharmacological interventions. It can be speculated that such non-immediate clinical effects are perhaps not routinely taken into consideration when selecting stroke patients for echocardiography, leading to the observed motley mix of TTE rates in our cohort. The pressure to reduce the length of in-hospital stay in Germany may have further contributed to this finding.

Interpreting our observation of significantly higher TTE rates in pSCs than cSCs remains challenging. It could be speculated that cSCs may, due to high patient volumes and restricted resources, mainly perform hyper-acute stroke treatments (e.g. thrombectomy) and delegate further cardiac work-up (including echocardiography) to pSCs or ambulatory care.

Interestingly, highest TEE rates were achieved at departments having a cardiological department on site, underlining the importance of an interdisciplinary approach for treating patients with acute stroke.

Limited resources are most likely responsible for our observation that increasing numbers of patients lead to decreasing TTE and TEE rates; for TEE mediated in particular by cSCs. In the context of finite resources, it is of note, that our data suggest that a considerable number of stroke units do not conduct any TTE in a substantial number of patients. It would hence be important to include more detailed recommendations with respect to echocardiography in future guidelines. Whether higher proportions of echocardiographic work-up may lead to better clinical long-term outcome should be focus of further evaluations.

Our study has several strengths and limitations. Our results are confined to stroke units in Germany and we cannot draw conclusions regarding non-industrialized countries. However, the large number of included departments provides a representative picture on the use of echocardiography in acute stroke centers and we assume similar findings in other European countries. It is to note that the “real” rate of combined examinations could be larger with larger overlap if more than one examination would have been performed per patient. Moreover, due to the study design, our data also include stroke patients with ICH, usually not undergoing echocardiography in clinical practice, but the overall number of them was low (according to a random sample 5–6%). All the more, as individual patient data are not available, we are not able to include e.g. age or gender aspects or information on stroke etiology or stroke subgroups into the current analysis.

## Conclusion

Heterogeneous TTE rates reflect ambiguous guideline recommendations on echocardiography in stroke patients and limited resources may explain decreasing TTE and TEE rates in departments with high patient volumes. Moreover, a considerably number of stroke units do not perform echocardiography in a relevant proportion of patients.

More detailed recommendations with respect to echocardiography in future guidelines and prospective studies evaluating the impact of echocardiographic examinations on long-term prognosis in stroke patients are warranted.

## Supplementary Information

**Additional file 1: Table S1.** Reported and calculated variables with regard to the year of audit and respective differences between years (N = number; *Chi^2^-Test; $ Kruskal-Wallis Test; IQR = Interquartile range).** Table S2A**. Associations between quartalized TTE rates (%) and the existence of a cardiological department for the overall data set, comprehensive stroke centers only and primary stroke centers only respectively. † U-Test.** Table S2B**. Associations between quartalized TEE rates (%) and the existence of a cardiological department for the overall data set, comprehensive stroke centers only and primary stroke centers only respectively. † U-Test

## Data Availability

The dataset used and analyzed during the current study are available from the corresponding author on reasonable request.

## Abbreviations

TTE: Transthoracic echocardiography
TEE: Transesophageal echocardiography
pSC: Primary stroke center
cSC: Comprehensive stroke center
TIA: Transient ischemic attack
ECG: Electrocardiography
IQR: Interquartile range
N: Number
OR: Odds ratio
CI: Confidence interval
ESO: European stroke organization
ICH: Intracerebral hemorrhage
