# Supplementary material for: Echocardiography in acute stroke patients: a nationwide analysis in departments with certified stroke units in Germany
Source: Neurol Res Pract. 2023 Jan 19;5:3. doi: 10.1186/s42466-022-00229-1 (PMC9850591; doi:10.1186/s42466-022-00229-1)
Supplement: Supplementary file 1 — Additional file 1: Table S1. Reported and calculated variables with regard to the year of audit and respective differences between years (N = number; *Chi2-Test; $ Kruskal-Wallis Test; IQR = Interquartile range). Table S2A. Associations between quartalized TTE rates (%) and the existence of a cardiological department for the overall data set, comprehensive stroke centers only and primary stroke centers only respectively. † U-Test. Table S2B. Associations between quartalized TEE rates (%) and the existence of a cardiological department for the overall data set, comprehensive stroke centers only and primary stroke centers only respectively. † U-Test [file 42466_2022_229_MOESM1_ESM.pdf]

ADDITIONAL FILE 1

Table S1: Reported and calculated variables with regard to the year of audit and respective differences between years **(**N = number; *Chi²-Test; $ Kruskal-Wallis Test; IQR = Interquartile range).

	**Variable**	**Total** (one audit cycle)	**Audit year 2018**	**Audit year 2019**	**Audit year 2020**	**p**
Reported Variables (included departments)	Comprehensive stroke centers: N (%)	132 (42.6)	43 (41.0)	43 (41.7)	46 (45.1)	0.816*
	Primary stroke centers: N (%)	178 (57.4)	62 (59.0)	62 (59.0)	60 (58.3)	
	Neurological department existent: N (%)	301 (97.1)	103 (98.1)	100 (97.1)	98 (96.1)	0.688*
	Cardiological department existent: N (%)	230 (74.2)	75 (71.4)	77 (74.8)	78 (76.5)	0.700*
	Number of Stroke Unit beds: N (range)	8 (4-22) IQR: 6-10	8 (4-20)	8 (4-22)	8 (4-16)	0.685$
	Length of hospital stay: Days (median, IQR)	7.5 (6.6-8.8)	7.9 (6.6-9.1)	7.4 (6.5-8.6)	7.5 (6.7-8.6)	0.148$
	Departmental number of stroke patients: median (IQR)	784 (583-1043)	761 (570-1136)	804 (588-1020)	811 (600-992)	0.424$
	Number of TTEs: median (IQR)	455 (269-645)	503 (279-701)	400 (227-638)	517 (360-612)	0.074$
	Number of TEEs: median (IQR)	170 (121-243)	180 (118-261(	184 (118-244)	154 (125-216)	0.192$
Calculated Variable (included departments)	TTE rate (%): median (IQR)	63.3 (39.0-80.8)	64.0 (40.9-79.8)	60.7 (34.5-79.6)	67.0 (44.6-83.6)	0.533$
	TEE rate (%): median (IQR)	21.7 (16.4-29.5)	20.3 (15.7-31.2)	22.0 (16.5-30.3)	20.6 (17.1-27.9)	0.969$
	Rates of patients with no-,or combined examinations (%) [TTErate+TEErate)-100]: median (IQR)	-13.6 (-33.5 – 5.1)	-13.6 (-29.0-2.6)	-18.4 (-43.2 – 4.2)	-8.4 (-31.7 – 7.6)	0.505$

Table S2A: Associations between quartalized TTE rates (%) and the existence of a cardiological department for the overall data set, comprehensive stroke centers only and primary stroke centers only respectively. † U-Test

All included stroke units			
	**With cardiological department (N=230)**	**Without cardiological department (N=80)**	**p†**
TTE rate (median, IQR)	63.1 (38.3-80.4)	67.3 (42.9-82.2)	0.356
TTE rate, 1st quartile (median, IQR), N	23.7 (15.5-32.4), 58	24.5 (16.2-30.6), 18	0.788
TTE rate 2nd quartile (median, IQR), N	52.5 (44.9-58.4), 59	50.6 (47.0-57.8), 19	0.884
TTE rate 3rd quartile (median, IQR), N	72.5 (67.7-77.3), 58	74.3 (69.8-78.0), 21	0.369
TTE rate 4th quartile (median, IQR), N	91.3 (85.1-95.4), 55	92.4 (85.7-97.4), 22	0.652
**Comprehensive stroke centers only**			
	**With cardiological department (N=117)**	**Without cardiological department (N=15)**	**p†**
TTE rate (median, IQR), N	56.7 (33.4-75.0)	74.8 (31.9-80.7)	0.420
TTE rate, 1st quartile (median, IQR), N	25.2 (13.3-31.9), 35	28.7 (15.9-31.5), 4	0.772
TTE rate 2nd quartile (median, IQR), N	53.4 (45.0-60.0), 35	47.0 (40.75-n/a), 3	0.607
TTE rate 3rd quartile (median, IQR), N	70.7 (66.2-75.7), 25	78.2 (74.9-80.2), 5	**0.022**
TTE rate 4th quartile (median, IQR), N	92.2 (83.8-97.1), 22	84.8 (81.5-n/a), 3	0.723
**Primary stroke centers only**			
	**With cardiological department (N= 113)**	**Without ccardiological department (N=65)**	**p†**
TTE rate (median, IQR), N	70.2 (42.8-84.4), 113	66.9 (44.8-84.5), 65	0.831
TTE rate, 1st quartile (median, IQR), N	23.6 (18.7-37.0), 23	22.6 (16.2-30.2), 14	0.434
TTE rate 2nd quartile (median, IQR), N	51.5 (43.6-58.0), 24	52.3 (47.6-57.6), 16	0.423
TTE rate 3rd quartile (median, IQR), N	74.5 (70.0-78.1), 33	73.7 (68.3-76.9), 16	0.551
TTE rate 4th quartile (median, IQR), N	90.5 (85.9-94.8), 33	93.0 (86.5-97.3), 19	0.476

Table S2B: Associations between quartalized TEE rates (%) and the existence of a cardiological department for the overall data set, comprehensive stroke centers only and primary stroke centers only respectively. † U-Test

All included stroke units			
	**With cardiological department (N=230)**	**Without cardiological department (N=80)**	**p†**
TEE rate (median, IQR), N	21.5 (16.2-29.4)	20.1 (16.5-30.5)	0.930
TEE rate, 1st quartile (median, IQR), N	14.3 (12.3-15.4), 59	14.1 (12.4-15.6), 18	0.962
TEE rate 2nd quartile (median, IQR), N	18.9 (16.4-20.1), 54	16.5 (17.0-19.6), 24	0.183
TEE rate 3rd quartile (median, IQR), N	25.1 (23.1-27.1), 61	23.9 (21.6-27.3), 17	0.336
TEE rate 4th quartile (median, IQR), N	37.0 (31.6-42.0), 56	40.1 (31.8-45.7), 21	0.485
**Comprehensive stroke centers only**			
	**With cardiological department (N=117)**	**Without cardiological department (N=15)**	**p†**
TEE rate (median, IQR), N	20.4 (16.1-28.0), 117	27.9 (20.0-31.6), 15	0.063
TEE rate, 1st quartile (median, IQR), N	14.3 (11.5-15.4), 32	0	n/a
TEE rate 2nd quartile (median, IQR), N	18.6 (17.4-19.5), 28	18.0 (16.6-19.8), 4	0.531
TEE rate 3rd quartile (median, IQR), N	25.5 (23.5-27.8), 33	22.8 (21.7-26.9), 4	0.154
TEE rate 4th quartile (median, IQR), N	36.3 (32.5-41.6), 24	31.6 (30.5-32.7), 7	**0.026**
**Primary stroke centers only**			
	**With cardiological department (N=113)**	**Without cardiological department (N=65)**	**p†**
TEE rate (median, IQR), N	21.6 (16.6-30.4)	19.5 (16.0-28.0)	0.207
TEE rate, 1st quartile (median, IQR), N	14.7 (13.5-15.7), 27	14.1 (12.4-15.6), 18	0.694
TEE rate 2nd quartile (median, IQR), N	19.4 (18.4-20.4), 26	18.6 (17.1-19.6), 20	0.057
TEE rate 3rd quartile (median, IQR), N	24.4 (22.2-27.0), 28	25.3 (21.7-27.3), 13	0.989
TEE rate 4th quartile (median, IQR), N	37.4 (31.2-44.7), 32	41.8 (39.1-48.8), 14	**0.042**
